# Rapid multiplex liver gene-editing in mice using adeno-associated virus 8 or lipid nanoparticles

**DOI:** 10.1016/j.omta.2026.201720

**Published:** 2026-03-18

**Authors:** Dandan Wu, Isabelle Bolt, Dagmar W. Tolenaars, Suzanne Duijst, Wietse In het Panhuis, Stijn R.J. Hofstraat, Robby Zwolsman, Roy van der Meel, Coen C. Paulusma, Stan F.J. van de Graaf

**Affiliations:** 1 Tytgat Institute for Liver and Intestinal Research, Amsterdam University Medical Centers, University of Amsterdam, Meibergdreef 69, 1105 BK Amsterdam, the Netherlands; 2 Amsterdam Gastroenterology, Endocrinology Metabolism (AGEM), Amsterdam University Medical Centers, Meibergdreef 9, 1105 AZ Amsterdam, the Netherlands; 3 Laboratory of Chemical Biology, Department of Biomedical Engineering, and Institute for Complex Molecular Systems, Eindhoven University of Technology, P.O. Box 513, 5600 MB Eindhoven, the Netherlands

**Keywords:** bile salt conjugation, rapid gene-editing, tyrosinemia, cholestasis, more humanized mice model

## Abstract

Somatic liver knockout (SLiK) is a method developed to rapidly generate liver-specific knockout of one or several genes. However, the original protocol relies on hydrodynamic tail vein injection (HTVI), a procedure associated with low transfection efficiency and animal discomfort due to cardiac stress. To address these challenges, we evaluated whether HTVI could be adapted to adeno-associated virus serotype 8 (AAV8) or lipid nanoparticles (LNPs). In addition, we sought to expand SLiK functionality by incorporating transgene overexpression together with gene knockout. Two AAV8 vectors were co-injected into *Fah*^−/−^*/spCas9*^Tg^ mice, each carrying one gRNA targeting *Hpd* and a second gRNA targeting gene of interest (GOI), together with a cytomegalovirus (CMV)-driven transgene cassette. This design enables dual knockout with concurrent overexpression in the same hepatocyte. LNPs were tested as an alternative non-viral method to deliver individual gRNA. Both AAV8- and LNP-mediated approach achieved near-complete *Hpd* inactivation within 2 months, without significant adverse effects. Using AAV8, we generated *Cyp2c70* knockout mice that recapitulated the known bile-acid phenotype. We also established mice models with either humanized NTCP (*SLC10A1*) or AGXT by deleting the murine genes and overexpressing their human ortholog. Our platform offers a versatile strategy for rapid, multiplex hepatocyte gene editing for broader scientific community.

## Graphical abstract

**Figure:**
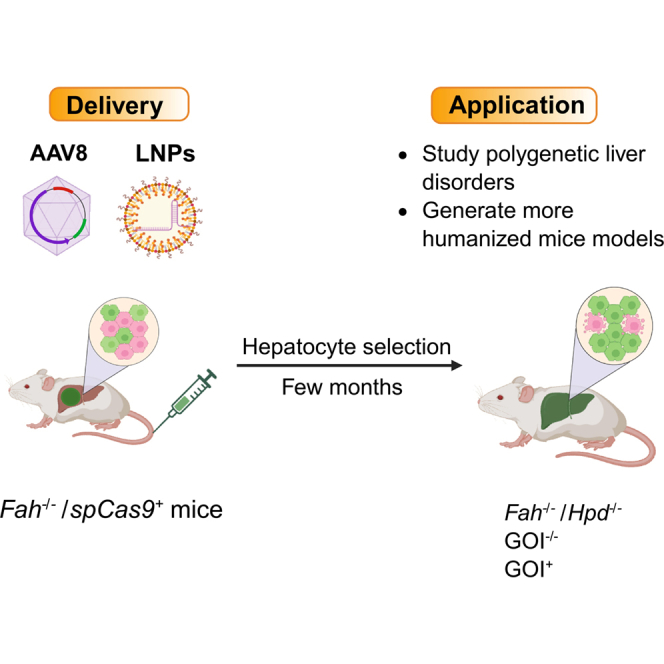

## Teaser

Van de Graaf and colleagues present a CRISPR–Cas9-based platform that enables rapid and multiplex hepatocyte editing in adult mice using AAV8 or LNPs. The platform allows for hepatocyte-specific gene knockout with concurrent transgene overexpression within few months.

## Introduction

**Figure 1:**
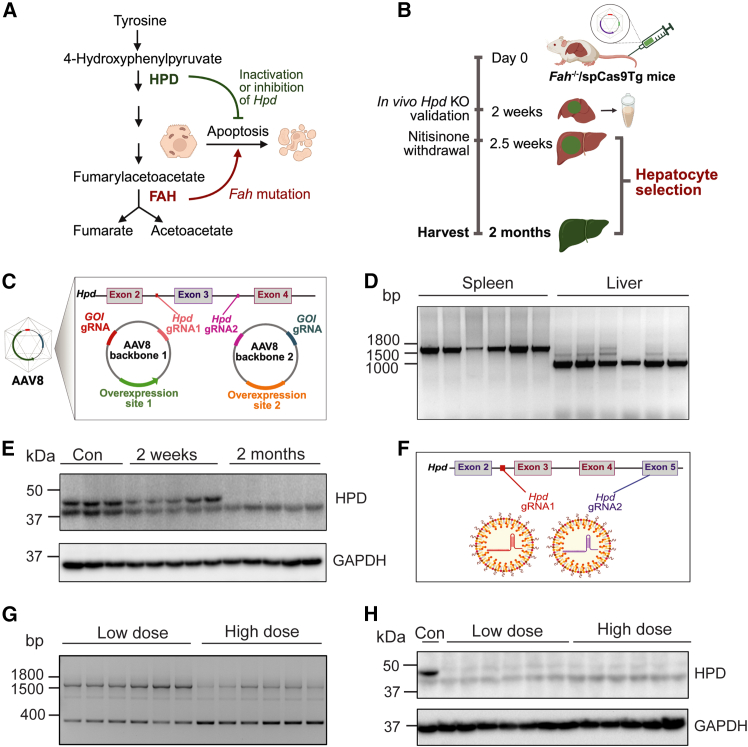
Efficient *Hpd* inactivation achieved by both AAV8- and LNP-mediated SLiK as alternatives to hydrodynamic tail vein injection. (A) Schematic representation of the reversal of *Fah*-deficiency-mediated toxicity through *Hpd* inactivation. (B–E) AAV8-mediated SLiK. (B) Experimental design. Nitisinone was used as an HPD inhibitor prior to hepatocyte selection. (C) Schematic overview of plasmid design. (D) Genomic PCR for *Hpd* excision in liver (*n* = 6), with spleen as a genomic control for *Hpd* excision. The PCR on spleen indicates that unexcised *Hpd* is around 1,500–1,800 bp, whereas a ∼1,000 bp band was detected in the liver, indicating liver-specific Hpd excision. (E) Western blot for HPD in liver lysates (left to right: *Fah*^±^ controls [*n* = 2]; *Fah*^−/−^ mice at 2 weeks post-AAV injection [*n* = 5]; *Fah*^−/−^ mice at 2 months after AAV injection [*n* = 5]). (F–H) LNP-mediated SLiK. (F) LNP and gRNA design. (G) Genomic PCR of hepatic *Hpd* excision in *Fah*^−/−^ mice treated with low-dose (0.05 mg/kg, *n* = 6) and/or high-dose LNPs (0.5 mg/kg, *n* = 6). (H) Western blot of hepatic HPD from LNP-treated mice. AAV8, adeno-associated virus 8; FAH, fumarylacetoacetate hydrolase; GAPDH, glyceraldehyde 3-phosphate dehydrogenase; HPD, 4-hydroxyphenylpyruvate dioxygenase; LNP, lipid nanoparticle; SLiK, somatic liver knockout. (A–C) and (F) are created using BioRender.

Despite advances in gene-editing, generating liver-specific mouse models targeting multiple genes remains time-consuming and costly.^1^ Moreover, growth advantages of unedited hepatocytes can lead to reduced editing efficiency over time.^2^ To overcome these limitations, Pankowicz et al. developed somatic liver knockout (SLiK)—a rapid method for knocking out multiple genes in livers of adult mice.^3^^,^^4^ The SLiK system leverages the selection pressure conferred by metabolites of tyrosine catabolism. Fumarylacetoacetate hydrolase (FAH) deficiency provides a growth disadvantage to unedited hepatocytes due to hepatotoxic accumulation of fumarylacetoacetate. However, inactivation of the upstream 4-hydroxyphenylpyruvate dioxygenase (HPD) using CRISPR-mediated knockout of *Hpd*, alone or in combination with guide RNAs (gRNAs) targeting other genes of interest (GOIs), rescues this phenotype, granting a proliferative advantage to edited hepatocytes^5^ (Figure 1A). Prior to gene-editing, mice are provided with drinking water containing the HPD inhibitor nitisinone to prevent hepatocellular toxicity.

The original SLiK protocol relies on hydrodynamic tail vein injection (HTVI), a delivery method that is not widely adopted due to its modest initial editing efficiency and the risk of cardiac complications in mice.^6^ These limitations restrict its broader implementation, and the approach requires substantial proliferation of edited hepatocytes to overcome loss of HPD-positive cells, potentially interfering with biological processes. To circumvent this challenge, we here substituted HTVI with milder and more efficient delivery systems, namely adeno-associated virus 8 (AAV8) and lipid nanoparticles (LNPs).^7^ Furthermore, we refined the plasmid design, enabling simultaneous gene knockout and transgene overexpression. We validated this system in a comprehensive set of five distinct experiments.

## Results

### Both AAV8-mediated and LNP-mediated SLiK successfully achieved *Hpd* knockout in mice within 2 months

First, we evaluated delivery efficiency and safety. We generated *Fah*^−/−^*/spCas9*^Tg^ mice, which constitutively express spCas9, and injected them with two AAV8 vectors (5∗10^12^ vg/kg for each vector) (Figures 1B and 1C). Each vector carried two gRNAs driven by a U6 promoter: one targeting an intron of *Hpd* and another targeting an early exon of a GOI. Because deletion of *Hpd* requires uptake of both AAV8 vectors, only hepatocytes transduced by both vectors gain the selection advantage in *Fah*^−/−^ mice. This co-delivery ensures that GOI knockout occurs in the same population of edited cells. Each AAV8 vector also allows cytomegalovirus (CMV)-promoter-driven overexpression of any cDNA compatible with AAV packaging. Two months post-injection, we observed complete and liver-specific *Hpd* knockout of treated mice. Genomic PCR confirmed excision of *Hpd* exon 3 in the liver, but not in the spleen and skeletal muscle (Figures 1D and S2A), indicating hepatic specificity. Western blot analysis showed complete loss of HPD protein 2 months post-injection (Figure 1E). Body weights remained stable (Figure S2B), and liver injury markers aspartate aminotransferase (AST), alanine transaminase (ALT), and alkaline phosphatase (ALP) showed only mild elevations compared to controls (Figures S2C–S2E).

Second, we tested LNPs as an alternative delivery vehicle at low (0.05 mg/kg) and high (0.5 mg/kg) doses (Figure 1F). Both doses induced near-complete *Hpd* knockout within 2 months at the genomic (Figure 1G) and protein level (Figure 1H), without significant weight loss or notable changes in liver toxicity markers (Figures S2F–S2I). These results demonstrate that both AAV8 and LNPs are effective and well-tolerated alternatives to HTVI for inducing *Hpd* inactivation in SLiK system.

### The AAV8-mediated SLiK system enables efficient knockout of one GOI

**Figure 2:**
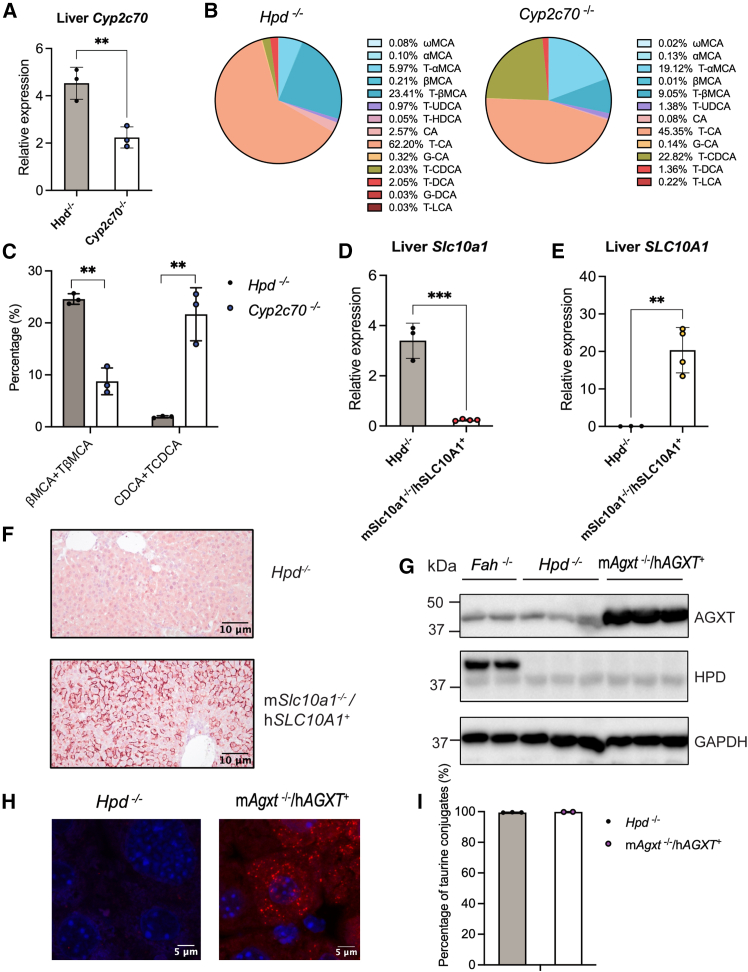
AAV8-mediated SLiK enables multiplex gene-editing in the liver. (A–C) Male mice were injected with AAV8 to knockout *Cyp2c70* (*Cyp2c70*^−/−^, *n* = 3) versus control (*Hpd*^−/−^, *n* = 3). (A) Relative mRNA expression of *Cyp2c70* in liver tissue. (B) Bile salt composition in bile. (C) Quantification of MCAs and CDCAs in bile. (D–F) Male mice were injected with AAV8 to knockout murine *Slc10a1* (encoding NTCP) and overexpress human *SLC10A1* (*mSlc10a1*^−/−^/*hSLC10A1*^+^, *n* = 4) versus control (*Hpd*^−/−^, *n* = 3). (D) Relative mRNA expression of murine *Slc10a1* in liver tissue. (E) Relative mRNA expression of human *SLC10A1* in liver tissue. (F) Immunohistochemical staining of human NTCP in liver tissue. (G–I) Male mice were injected with AAV8 to knockout *Agxt* and overexpress human *AGXT* (m*Agxt*^−/−^/h*AGXT*^+^, *n* = 3) or control (*Hpd*^−/−^, *n* = 3). (G) Western blot of HPD and AGXT. (H) Immunofluorescence staining of AGXT in liver sections. Nuclei are stained with DAPI (blue), and AGXT is visualized using Alexa Fluor 594 (red). (I) Quantification of bile salt conjugation ratio (taurine- vs. glycine-conjugated species) based on five major bile salts (CA, UDCA, CDCA, DCA, and LCA). For (A–C): analyzed 3 months post-AAV8 injection; For (D–F): analyzed 1 month post-AAV8 injection. For (G–I): analyzed 3 months post-AAV8 injection. Bars represent means ± SD. ∗*p* < 0.05, ∗∗*p* < 0.01, *p* < 0.001, according to unpaired Student’s *t* test. AAV8, adeno-associated virus 8; AGXT, alanine-glyoxylate aminotransferase; CYP2C70, cytochrome P450 2C70; FAH, fumarylacetoacetate hydrolase; GAPDH, glyceraldehyde 3-phosphate dehydrogenase; HPD, 4-hydroxyphenylpyruvate dioxygenase; NTCP, sodium/taurocholate co-transporting polypeptide; SLC10A1, solute carrier family 10 member 1; SLiK, somatic liver knockout.

Third, we introduced inactivation of a GOI-targeting gRNA. Unlike mice, humans lack muricholic acids (MCAs) and produce higher levels of chenodeoxycholic acid (CDCA). Cytochrome P450 2C70 (*Cyp2c70*) encodes the enzyme responsible for converting CDCA into MCAs in mice. The deletion of *Cyp2c70* is known to humanize the bile salt profile in mice, but when present from birth, it leads to severe liver damage.^8^ Using AAV8-mediated SLiK, we generated liver-specific *Cyp2c70* knockout mice. qPCR showed a ∼50% reduction of *Cyp2c70* mRNA (Figures 2A and S3A). Bile salt profiling revealed decreased taurine-conjugated β-muricholic acid (β-MCA) from 23.4% to 9.1%, accompanied by increased taurine-conjugated chenodeoxycholic acid (CDCA) from 2.0% to 22.8% in bile (Figures 2B and 2C; data of female mice is depicted in Figures S3B and S3C). Notably, the true knockout efficiency is likely higher than suggested by qPCR, as the bile acid composition closely resembled that of conventional liver-specific knockout mice, which exhibit ∼95% reduction of CYP2C70 protein.^8^ Together, these data suggested AAV8-SLiK provides an efficient strategy to generate mice with a more human-like, hydrophobic bile salt pool.

### The AAV8-mediated SLiK system enables simultaneous knockout of one GOI and overexpression of another GOI

Fourth, to assess whether AAV8-mediated SLiK can achieve simultaneous gene knockout and transgene overexpression, we performed a proof-of-concept study targeting two genes. Na^+^-taurocholate co-transporting polypeptide (NTCP, encoded by *SLC10A1*) is a liver-specific bile salt transporter. Given the differences between human and murine NTCP protein structures, expressing human *SLC10A1* in mice may facilitate the translation of preclinical findings. We generated human-NTCP-expressing mice by deleting endogenous *Slc10a1* (*mSlc10a1*) and overexpressing human *SLC10A1* (*hSLC10A1*). qPCR revealed a reduction in *mSlc10a1* and induction of *hSLC10A1* expression (Figures 2D and 2E). Immunohistochemistry confirmed hNTCP protein abundance in liver tissue (Figure 2F). These findings demonstrate that AAV-modified SLiK enables concurrent gene knockout and transgene overexpression.

### The AAV8-mediated SLiK system allows for the investigation of new biological hypotheses

We next applied this platform to test a long-standing hypothesis regarding species-specific bile acid conjugation. Whereas mice predominantly conjugate bile acids with taurine, humans mainly display glycine conjugation. Alanine-glyoxylate aminotransferase (AGXT) has been proposed to contribute to this divergence. Human AGXT is localized in peroxisomes, where it could contribute to high local concentrations of glycine, whereas mouse AGXT is a cytosolic protein.^9^ We therefore examined the impact of AGXT manipulation on conjugation patterns in mice. To experimentally challenge this hypothesis, we used this system to knock out *mAgxt* and overexpress *hAGXT*. Sanger sequencing of liver genomic DNA confirmed on-target editing at the m*Agxt* locus (Figure S3D). TIDE analysis was performed to estimate the editing efficiency from the Sanger sequencing data,^10^ revealing an average editing efficiency of approximately 40% (Figure S3E). To further quantify editing efficiency in hepatocytes, PCR amplicons spanning the m*Agxt* gRNA target site were TA-cloned from whole-liver genomic DNA and subjected to Sanger sequencing. Among 47 colonies derived from four mice, 42% of alleles were wild-type, whereas 58% carried mutations, the majority of which were frameshift indels. Considering that hepatocytes account for approximately 60%–70% of total liver cells (and ∼80% of liver volume), these results indicated a high editing efficiency of m*Agxt* in hepatocytes. mRNA levels of m*Agxt* were comparable between m*Agxt*^−/−^/h*AGXT*^+^ mice and controls (Figures S3F and S3G). Overexpression of hAGXT was confirmed at the protein level in male mice (Figure 2G), but not in females (Figure S3H), in accordance with lower AAV transduction efficiency in female mice.^11^ AGXT staining in livers of *mAgx*t^−/−^/*hAGXT*^*+*^ displayed a punctate, heterogeneous pattern, indicating a potential peroxisomal localization,^12^ while *Hpd*^−/−^ control animals display only weak cytosolic AGXT signal (Figure 2H). However, the proportion of taurine-conjugated bile salts remained unchanged between *hAGXT*-overexpressing and control mice (Figure 2I).

## Discussion

We present a rapid and efficient strategy for hepatocyte-specific gene deletion that couples CRISPR/Cas9 with *Hpd*-based selection, enabling near-complete liver repopulation within 2 months. Using AAV8, the platform enables simultaneous knockout of one or two GOIs in addition to *Hpd*. Moreover, the vector design supports concurrent overexpression of one or two transgenes within the same hepatocytes. Safety profiles were acceptable, as body weight remained stable and liver enzymes were only mildly increased at the end of study. Furthermore, no edits were detected at the top three predicted off-target sites by Sanger sequencing (not shown).

Originally, the SLiK system relied on HTVI for *in vivo* delivery. Although HTVI is a cost-effective and rapid method for plasmid DNA delivery and accommodates multiple sgRNAs for multiplex genome editing, it requires high-volume injection (8–10% body weight) that induces substantial hemodynamic stress.^13^ As a result, HTVI is incompatible with modern animal welfare frameworks, especially those strictly enforced in the European Union. To address these limitations, we adapted the SLiK platform for AAV8- and LNP-based delivery. These systems represent the current translational standard in gene therapy and offers a substantially improved safety profile.^14^ Through vector optimization, we demonstrated that our AAV8-based SLiK maintains high efficiency for multiplex genome editing. However, AAV-based delivery is intrinsically constrained by limited packaging capacity and immunogenicity.^14^ To mitigate this, we further explored LNPs as a non-viral alternative in a proof-of-concept setting. LNPs can accommodate larger genetic payloads and allow for re-administration, making them especially relevant for treating diseases requiring chronic dosing. Collectively, these delivery strategies expand the applicability of the SLiK system while aligning with contemporary standards for safety and translational relevance.

A key strength of the optimized SLiK platform is its ability to couple endogenous gene knockout with concurrent transgene overexpression within the same hepatocyte. This design enables the rapid generation of mice that lack specific murine proteins while expressing their human orthologs, thereby offering several important translational applications. First, it enables the precise investigation of liver-specific genes (single or dual targets) to elucidate novel mechanisms in hepatic disease pathology. Second, for ubiquitously expressed genes, SLiK allows researchers to dissect the liver’s specific contribution to systemic metabolic regulation and drug metabolism. Third, and perhaps most significantly, this platform accelerates the generation of more “humanized” mouse models. By replacing endogenous mouse proteins with human orthologs, SLiK provides a robust preclinical platform for testing human-specific therapeutics, such as small molecules or siRNAs that do not cross-react with murine targets prior to clinical trials, as demonstrated by our proof-of-concept studies with NTCP. This approach substantially shortens timelines and reduces the costs associated with traditional transgenic breeding, allowing for the rapid biological validation of therapeutic hypotheses. Beyond disease modeling, SLiK also offers a powerful framework for precision medicine by enabling rapid functional assessment of clinical variants of unknown significance (VUS). Patient-specific mutations can be introduced and evaluated on a null background, allowing pathogenic variants to be distinguished from benign polymorphisms in a physiologically relevant *in vivo* context.

We further utilized this platform to examine the role of AGXT in bile acid conjugation. To this end, we knocked out murine *Agxt* and overexpressed human *AGXT* in hepatocytes. Western blot confirmed hAGXT overexpression, and Sanger sequencing of liver genomic DNA verified on-target editing at the *mAgxt* locus. A limitation of this study is that due to high coding-sequence homology, available antibodies cannot distinguish mouse from human AGXT, and m*Agxt* mRNA expression was not downregulated despite genomic edits. Nevertheless, these data indicate that hAGXT expression alone does not shift bile acid conjugation from taurine- to glycine-dominant forms in mice, suggesting that additional factors may govern species-specific conjugation.

In the same study, we observed that hAGXT overexpression was successfully induced in male mice but not in female mice using the AAV8-based system. This finding is consistent with previous reports demonstrating that AAV8-mediated liver transduction is androgen-dependent, with 5- to 13-fold higher transduction efficiency in male mice than in females.^15^ We speculate that LNP-based delivery may help overcome this sex-based limitation, as LNPs typically rely on ApoE-mediated endocytosis and do not exhibit the pronounced sex bias reported for AAV8,^16^ although this still require experimental validation in the future.

In the liver, regeneration response depends on the extent of loss: up to ∼30% (one-third) hepatectomy is restored primarily through hypertrophy, whereas larger hepatectomies involve substantial hyperplasia.^17^ The reported transfection efficiency of HTVI is only ∼20%–40%,^18^ implying that a large fraction of the liver must undergo regeneration to recover its original mass. In contrast, in our study we achieved 60%–70% *Hpd* editing prior to selection, suggesting that subsequent liver repopulation was driven predominantly by hypertrophy rather than hyperplasia. This regenerative profile likely minimizes activation of proliferative pathways that could otherwise confound interpretation of metabolic phenotypes.

A recent study by Chen et al. reported that CRISPR/Cas9 gene therapy increased the risk of hepatocellular cancer 12 months post-treatment in the *Fah*^−/−^ mouse model.^18^ Although no liver tumors were observed at 3 months post-treatment in this study, surveillance and histopathological evaluation may be warranted also when using this system in extended experiments.

While our AAV vector design allows simultaneous deletion and transgene expression, single-exon targeting may be insufficient for genes with redundancy or alternative isoforms. In such cases, dual-guide strategies targeting two exons or functionally homologous isoforms may improve knockout efficiency. Moreover, since CMV-driven transgene overexpression is non-integrating and likely to decline over time,^19^ durability should be quantified in longer studies.

Despite these considerations, our AAV8/LNP-modified SLiK platform is a versatile and flexible tool for rapidly generating advanced mouse models for biomedical research.

## Materials and methods

### gRNA design

A panel of gRNAs targeting introns of *Hpd* and early exons of GOIs were designed using the Synthego design tool (https://design.synthego.com/#/). Candidate gRNAs with high predicted performance scores were further evaluated using the IDT CRISPR-Cas9 gRNA Checker to eliminate sequences with high predicted off-target potential or low on-target efficiency (https://www.idtdna.com/site/order/designtool/index/CRISPR_SEQUENCE). The final selected gRNA sequences are listed in Table 1.

**Table 1:** Selected gRNA sequences and their corresponding genomic target regions

Gene name	gRNA name	gRNA sequence (5’ – 3′)	Targeting location
*Hpd*	*Hpd* gRNA 1	AGCCCAAAACCTCCGAGAAT	intron 3
*Hpd*	*Hpd* gRNA 2	TGCAGCTTAGCCATACATCT	intron 4
*Slc10a1*	*mSlc10a1* gRNA	GAGGGGCATGATACCGTACT	exon 2
*Cyp2c70*	*Cyp2c70* gRNA	CATAGACCTTAAGACCATGA	exon 3
*Agxt*	*mAgxt* gRNA	GCCAAGGCCAGTGTGACGCT	exon 1

### Molecular cloning

For *Hpd* knockout generation, two plasmids were used. Plasmid 1 (AAV-Hpd1-GOI, VectorBuilder, VB211001-1008sqe) contains two U6-driven guide RNA (gRNA) cassettes and one CMV-driven multiple cloning site (MCS) cassette. A gRNA targeting the second intron of *Hpd* (Hpd 1) was pre-cloned into one of the gRNA cassettes. Plasmid 2 (AAV-Hpd2-GOI) was generated by replacing Hpd 1 with a gRNA targeting the third intron of *Hpd* (Hpd 2) in plasmid 1 using Gibson assembly (New England Biolabs, E55l0S). The sites for gRNA of GOI are occupied by non-functional stuffer sequence when they are not in use.

To generate *Cyp2c70* knockout mice, plasmid 1 [AAV-Hpd1-GOI (empty)] was used, and *Cyp2c70* gRNA was cloned into the GOI site of *Hpd* plasmid 2 (AAV-Hpd2-Cyp2c70).

For simultaneous knockout of murine *Slc10a1* and overexpression of human *SLC10A1*, plasmid 1 (VectorBuilder, VB221018-1569xue) containing one U6-driven Hpd gRNA1, one U6-driven *mSlc10a1* gRNA, and one CMV-driven *hSLC10A1* coding sequence was used. An HA-tag was added at the N-terminus of *the hSLC10A1* sequence to enable later detection by immunostaining. Plasmid 2 is AAV-Hpd2-GOI.

For *mAgxt* knockout and *hAGXT* overexpression, plasmid 1 (VectorBuilder, VB240220-1601uqb) containing one U6-driven *Hpd* gRNA1, one U6-driven *mAgxt* gRNA, and one CMV-driven *hAGXT* coding sequence was constructed by VectorBuilder. Plasmid 2 was used containing an empty GOI site [AAV-Hpd2-GOI (empty)].

### Adeno associated virus 8 production

Insertion of gRNAs was verified by Sanger sequencing prior to AAV production. AAV8 was produced by co-transfecting HEK293T cells with the target plasmid and the helper plasmid pDP8, as previously described.^20^ At 66 h post-transfection, cells were harvested, and AAV particles were purified using an iodixanol (OptiPrep; Sigma-Aldrich, 07820) gradient followed by concentration with Ultracel 100K centrifugal filter units (Amicon, UFC910024). Viral titers were determined from heat-inactivated, purified AAV preparations using RT-qPCR.

### Lipid nanoparticle production and characterization

LNPs containing single (s)gRNA (LNP-sgRNA) were produced via rapid mixing as described previously.^21^ The ionizable lipid ALC-0315 (SymoChem B.V.), cholesterol (Sigma-Aldrich), DSPC (Avanti Research, 850365), and DMG-PEG2000 (Avanti Research, 880151) were dissolved in ethanol at a molar ratio of 50:38.5:10:1.5 (ALC-0315:Cholesterol, DSPC, DMG-PEG2000). sgRNA stock solution was diluted in 25 mM sodium acetate (pH 4.0) to a concentration of 0.03 mg/mL. The aqueous and organic phases were mixed in a 3:1 volume-to-volume ratio and collected directly in a 12–14 kDa MWCO membrane (Spectra/Por) and dialyzed overnight (1× PBS, 4^o^C), with buffer refreshment after 4 h. Following dialysis, the product was filtered through a 0.2 μm syringe filter and kept sterile thereafter. Next, samples were concentrated using centrifugal filtration (100 kDa MWCO) and diluted to the desired RNA concentration and stored at 4^o^C until further use.

Hydrodynamic diameter and polydispersity index were determined with dynamic light scattering (DLS) using a Zetasizer Nano ZSP (Malvern Instruments). Samples were diluted 10-fold in PBS and measured three times with 10 runs of 10 s. RNA content was quantified using the Quant-it RiboGreen RNA assay (Thermo Fisher Scientific), as described previously.^22^ Briefly, LNP-sgRNA were dissolved in TE buffer and TE buffer supplemented with 2% Triton X-100 to disrupt particles and release encapsulated RNA. After adding 200 times diluted RiboGreen reagent, fluorescence was measured on a Tecan Spark microplate reader. The RNA encapsulation efficiency was calculated as the fraction sgRNA retained within the LNPs. LNP characteristics are displayed in Figure S1.

### Animal experiments

Experiments were approved by the National Committee for Animal Experiments of the Netherlands and by the Institutional Animal Care and Use Committee of the University of Amsterdam University Medical Center. All animal procedures conformed to the guidelines from Directive 2010/63/EU of the European Parliament on the protection of animals used for scientific purposes. Mice were housed in a temperature- and humidity-controlled facility under a 12-h cycle of light/dark cycle with *ad libitum* access to a standard chow diet and water unless stated otherwise. The *Fah*^−/−^*/*spCas9ˆTg strain was generated by crossing *Fah*^−/−^ mice and Rosa26-Cas9 knock-in mice (#026179; Jackson Laboratory). *Fah*^*+/−*^*/*spCas9ˆTg littermates were used as controls for validating the efficacy and safety of AAV8-mediated *Hpd* knockout. *Fah*^−/−^/spCas9ˆTg mice were maintained on nitisinone (7.5 mg/L) drinking water after weaning. AAV8 were administered by tail vein injection to both male and female mice (12–16 weeks old), except for the *mSlc10a1*^−/−^*/hSLC10A1*^*+*^ study, in which only males were used. A mixture of AAV8 encoding plasmid 1 and AAV8 encoding plasmid 2 (5 × 10^12^ vg/kg body weight for each AAV8) was used for each mouse. At 2.5 weeks post-injection, a subset of mice (*n* = 2/group) was euthanized to assess *Hpd* excision efficiency. In the initial AAV8-mediated *Hpd* inactivation study, excision efficiency reached approximately 60%–70%. Based on this result, nitisinone was subsequently withdrawn from the drinking water to initiate hepatocyte selection. Body weight was monitored twice weekly throughout the study period. After 8–14 weeks, mice were killed with CO_2_ around 4 h after the onset of the light phase.

### On-target and off-target effects

Potential off-target sites were predicted using the CCTop online tool (https://cctop.cos.uni-heidelberg.de/) by allowing up to four mismatches to the gRNA sequence. For each gRNA, the top three predicted off-target sites were selected for further validation. These genomic regions were PCR-amplified from mouse liver DNA, and the PCR products were gel-purified (Zymoclean Gel DNA Recovery Kit) and subjected to Sanger sequencing. A total of 15 candidate off-target sites across five distinct gRNAs were analyzed, and no off-target mutations were detected. On-target editing of *Cyp2c70* and m*Agxt* loci was assessed using the same approach. Sanger chromatograms revealed the presence of double peaks near the protospacer adjacent motif (PAM) regions, consistent with efficient on-target editing at the genomic level.

### TA cloning

PCR products amplified from whole-liver genomic DNA (the same amplicons used for Sanger sequencing of m*Agxt*) were cloned into the pCR2.1-TOPO vector (Invitrogen) according to the manufacturer’s instructions. Ligation reactions were transformed into electrocompetent *Escherichia coli*, and transformants were selected on LB agar plates containing ampicillin using blue-white screening. White colonies were picked and subjected to plasmid Sanger sequencing. A total of 47 white colonies from four mice were sequenced. The proportion of mutant versus wild-type alleles was used to estimate the overall hepatocyte editing efficiency in whole liver.

### Statistical methods

Data are presented as individual data points and/or means with standard deviation. Individual data points represent different mice. Results of two groups were compared with unpaired Student’s *t* test. One-way ANOVA followed by Tukey’s multiple comparisons was used when comparing more than two groups. *p* < 0.05 was considered statistically significant and is calculated using GraphPad Prism (v.10.2.0).

Additional experimental details are provided in the supplemental information.

## Data and code availability

Plasmid construct to make AAV8 will be shared via Addgene upon publication. Data are available to other researchers via contacting the corresponding author.

## Acknowledgments

This work was supported by 10.13039/501100003246Dutch Research Council (NWO) (grant no. Vici 09150182010007) (S.F.J.v.d.G.); Vidi 19681 (R.v.d.M.); and 10.13039/100016033Nederlandse Vereniging voor Gastroenterologie (S.F.J.v.d.G.). D.W. is supported by the 10.13039/501100004543China Scholarships Council (CSC: 202206230056). We sincerely thank Piter Bosma, Tulasi Yadati, and Elias Boer for the assistance with molecular experiments and Dirk R. de Waart for HPLC analyses.

## Author contributions

Conceptualization, S.F.J.v.d.G. Data curation, D.W. Formal analysis, D.W., I.B., and D.W.T. Funding acquisition, S.F.J.v.d.G. Investigation and interpretation, D.W., I.B., D.W.T., S.D., C.C.P., S.R.J.H., and R.Z. Methodology, S.F.J.v.d.G., C.C.P., and R.v.d.M. Supervision, S.F.J.v.d.G., C.P., W.P., and R.v.d.M. Visualization, D.W. Writing – original draft, D.W. and S.F.J.v.d.G. Writing – review and approval, all authors.

## Declaration of interests

The authors have no conflicts of interest to declare.

## Declaration of generative AI and AI-assisted technologies in the writing process

During the preparation of this work, the author(s) used ChatGPT-5 in order to check grammar and improve language. No AI tools were employed for data generation, data analysis, figure creation, or interpretation of scientific results. After using this tool/service, the authors reviewed and edited the content as needed and take full responsibility for the content of the publication.

## Supplemental information

Document S1. Figures S1–S3 and Tables S1 and S2

Document S2. Article plus supplemental information
