# Supplementary material for: Rapid multiplex liver gene-editing in mice using adeno-associated virus 8 or lipid nanoparticles
Source: Mol Ther Adv. 2026 Mar 18;34(2):201720. doi: 10.1016/j.omta.2026.201720 (PMC13148919; doi:10.1016/j.omta.2026.201720)
Supplement: Document S1. Figures S1–S3 and Tables S1 and S2 [file mmc1.pdf]

OMTA, Volume *34*

Supplemental information

Rapid multiplex liver gene-editing in mice using adeno-associated virus 8 or lipid nanoparticles

Dandan Wu, Isabelle Bolt, Dagmar W. Tolenaars, Suzanne Duijst, Wietse In het Panhuis, Stijn R.J. Hofstraat, Robby Zwolsman, Roy van der Meel, Coen C. Paulusma, and Stan F.J. van de Graaf

Supplemental material

Materials and Methods

Gene-editing and validation

Genomic PCR

Genomic DNA was extracted from mouse liver tissue using a standard proteinase K-based lysis protocol. Briefly, liver samples were lysed in buffer containing 100 mM Tris (pH 8.0), 200 mM NaCl, 5 mM EDTA, 0.2% SDS, and 100 µg/ml freshly added proteinase K at 55°C for overnight with shaking. DNA was precipitated with 2-propanol, washed with 70% ethanol, air-dried, and resuspended in TE buffer. Endpoint PCR was performed using primers flanking the CRISPR target sites to amplify the region of the *Hpd* locus (forward primer (5' to 3'): [CACCCATACTGTTCTCACGT]; reverse primer (5' to 3'): [TCAGCGAGGCATTAGCTATC]). PCR products were separated on a 1% agarose gel and visualized using a Bio-Rad Gel Doc imaging system.

qPCR

Total RNA was extracted from mouse liver using TRIzol reagent (Invitrogen, Waltham, USA), and RNA concentrations were quantified by spectrophotometry at a 260 nm using Nanodrop 1000 (Thermo Scientific, Waltham, MS). For each sample, 0.5 µg total RNA was treated with DNase I (Prometa, Madison, WI) and reverse transcribed into complementary DNA (cDNA) using oligo(dT) primers, random hexamers, and RevertAid reverse transcriptase (Thermo Scientific). qRT-PCR was performed using 2 µL of 1:5 diluted cDNA with SensiFAST SYBR No-ROX Kit (Bioline, London, UK) on a LightCycler 480 II system (Roche Diagnostics, Rotkreuz, Switzerland). Quantification of fluorescence signals was carried out using LinRegPCR software (version 2013.0, Academic Medical Center, Amsterdam, The Netherlands). Gene expression was normalized to the geometric mean of glyceraldehyde 3-phosphate dehydrogenase (*Gapdh*) and hypoxanthine phosphoribosyltransferase (*Hprt*). Primer sequences are listed in Table S1.

Western Blot

Liver tissues were homogenized in radioimmunoprecipitation assay (RIPA) lysis buffer (150 mM NaCl, 50 mM Tris pH 7.4, 5 mM EDTA, 1% Nonidet P40) supplemented with a protease inhibitors (Roche Diagnostics) using a TissueLyser (Qiagen) with a metal bead at 50 Hz for 10 minutes at 4 °C. Lysates were centrifuged at 14000 rpm for 15 minutes at 4 °C, and protein concentrations in the supernatant were determined by bicinchoninic acid assay (Thermo Scientific, Waltham, MA, USA). Equal amounts of protein (30ug) were separated by SDS-PAGE and transferred onto polyvinylidene difluoride (PVDF) membranes (Millipore, Burlington, MA, USA) using semi-dry blotting. Membranes were blocked with 5% non-fat milk in TBST for 90 minutes and incubated overnight at 4 °C with primary antibodies diluted in 5% non-fat milk/TBST (see Table S3 for antibody details and dilutions). After washing, membranes were incubated with horseradish peroxidase (HRP)-conjugated secondary antibodies (Table S2), and

signal detection was performed using enhanced chemiluminescence reagents (Lumi-Light, Roche Diagnostics) and visualized with an ChemicDoc MP Imaging System (BIO-RAD).

Immunohistochemistry for NTCP

Paraffin-embedded mouse liver tissues were sectioned (4.5 μm) using a Rotary Microtome (HM 340E, Thermo Scientific, Waltham, MS). Sections were stretched on glass slides and dried overnight at 37°C. Following deparaffinization, endogenous peroxidase activity was blocked by incubating the slides in 0.1% H_2O_2 in 100% methanol for 20 minutes. Antigen retrieval was performed by heating the sections at 120°C for 20 minutes in Tris-EDTA buffer (1 mM EDTA, 10 mM Tris, pH 9.0). Sections were then blocked for 20 minutes using Ultravision Protein Block (Thermo Scientific), and incubated overnight at 4°C with an anti-HA tag primary antibody (Sigma, H6908; 1:600 dilution in antibody diluent (ScyTek, Logan, UT)). The next day, sections were incubated with BrightVision anti-rabbit poly-HRP secondary antibody (Avantor, Radnor, PA), stained using red peroxidase substrate kit (Vector lab, Burlingame, CA) and counterstained with hematoxylin. Microscopy was performed using Olympus BX51 (Olympus Corporation, Tokyo, Japan) at a 20x objective.

Immunofluorescence for AGXT

Paraffin-embedded mouse liver sections were deparaffinized and rehydrated using Trilogy solution (Cell Marque, Rocklin, CA) for 40 minutes. Antigen retrieval was performed in Tris-EDTA buffer (1 mM EDTA, 10 mM Tris, pH 9.0). After blocking for 30 minutes with Ultravision Protein Block (Thermo Scientific, Waltham, MA), sections were incubated overnight at 4°C with a primary antibody against human/mouse AGXT (Sanbio, 22394-1-AP; 1:500 dilution in antibody diluent (ScyTek, Logan, UT)). The next day, sections were incubated for 1 hour at room temperature with a secondary antibody (anti-rabbit Alexa Fluor 594, Molecular Probes, A-11012; 1:100 in 1% BSA/TBST), protected from light. Slides were mounted using ProLong Gold Antifade Mountant (Invitrogen, Waltham, MA) on coverglass (Thermo Scientific). Z-stack imaging was performed using a Confocal Stellaris 8 microscope (Leica Microsystems, Wetzlar, Germany). Images were processed with histogram stretching using ImageJ v1.50i (NIH, Bethesda, MD).

Plasma biochemistry

For the measurement of liver injury biomarkers alanine transaminase (ALAT), aspartate transaminase (ASAT), and alkaline phosphatase (ALP), plasma samples were 4 times diluted in PBS and measured by routine clinical biochemistry testing at the Central Research Lab, Amsterdam Medical Center. These biomarkers are measured using photometric assay tests on the Roche Cobas c502/702 analyser (Roche Diagnostics).

Bile salt quantification

Bile salt concentrations in bile were measured by reverse-phase HPLC, which was an adaptation to the method used by Kunne et al [1]. For this purpose, plasma samples (10 μL) were deproteinized by addition of 5 volumes of acetonitrile (dropwise). Following centrifugation (10 minutes, 4°C, 14000g), solvent was

evaporated from supernatants and bile salts were solubilized in 200 μL 25% methanol. 100 μL sample was applied to a Hypersil C18 HPLC column (internal diameter: 3 μm, column length: 15 cm; Thermo Scientific, Breda, The Netherlands) operated at 20°C. The starting eluent consisted of 6.8 mM ammoniumformate (pH 4.2), followed by linear gradient or isocratic elution with acetonitrile at the indicated concentration: 0- 27% (0-1 minute), 27% (1-4 minutes), 27-29% (4-10 minutes), 29-32% (10-17 minutes), 32% (17-23 minutes), 32-36% (23-27 minutes), 36-45% (27-31 minutes), 45-52% (31-36 minutes), 52-76% (36-46 minutes), 76-100% (46-48 minutes), 100% (48-50 minutes), 100-0% (50-52 minutes), and 0% (52-58 minutes). The flow rate was 0.8 mL per minute. Detection was performed using a CAD (Charged Aerosol Detector). Quantification of the different bile salt species was performed by using a calibration curve for all different bile salt species.

Table S1. Primer sequences for quantitative reverse-transcription PCR

Target	Forward primer (5' to 3')	Reverse primer (5' to 3')
Mouse *Gapdh*	GACAACTCATCAAGATTGTCAGCA	TTCATGAGCCCTTCCACAATG
Mouse *Hprt*	CCTAAGATGAGCGCAAGTTGAA	CCACAGGACTAGAACACCTGCTAA
Mouse *Slc10a1*	TGGCTACCTCCTCCCTGATG	GCCAGGTTGTGTAGGAGGAT
Human *SLC10A1*	ATGCGCTATGTCATCAAGGGAG	AGGTGGCAATCAAGAGTGGT
Mouse *Cyp2c70*	AGTATGGCCCTGTGTTTACTGT	GCCTTGGCTGGTTCTACTGAG
Mouse Agxt	AAGGCATCCAGTATGTGTTCCA	TTCCGGTTAGAAAGGAGTCCC

Table S2. Primary and secondary antibodies used for immunoblotting

Antibody	Company	Reference number	Dilution
Anti-HPD	Sigma Life Science	HPA038322	1:1000
Anti-GAPDH	Proteintech	60004-1-Ig	1:1000
Anti-AGXT	Sanbio	22394-1 AP	1:1000
Anti-Rabbit IgG	BioRad	170-6515	1:5000
Anti-Mouse IgG	BioRad	170-6516	1:5000

Supplemental Figures

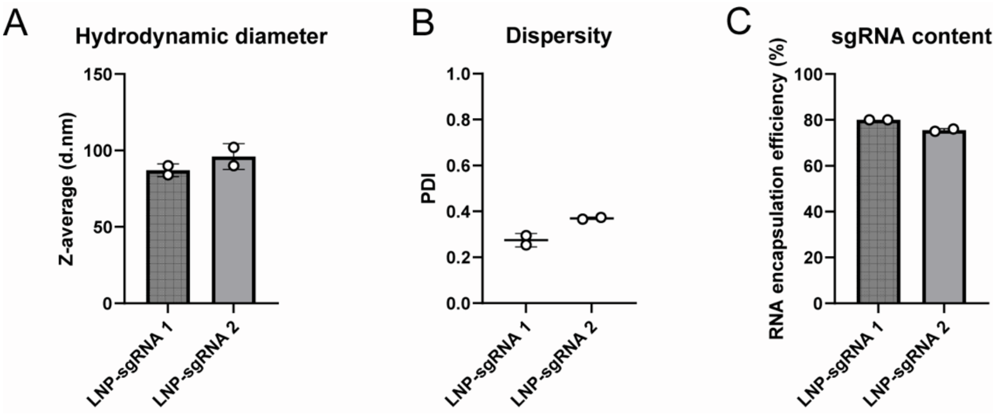

Figure S1. Physicochemical analysis of LNPs-sgRNA. LNPs containing two different sgRNAs were produced and characterized. (A-B) The LNPs' hydrodynamic diameter expressed as Z-average and PDI. (C) sgRNA encapsulation efficiency. Bars represent means ± SD. LNPs, lipid nanoparticles; PDI, polydispersity index; sgRNA, single guide RNA.

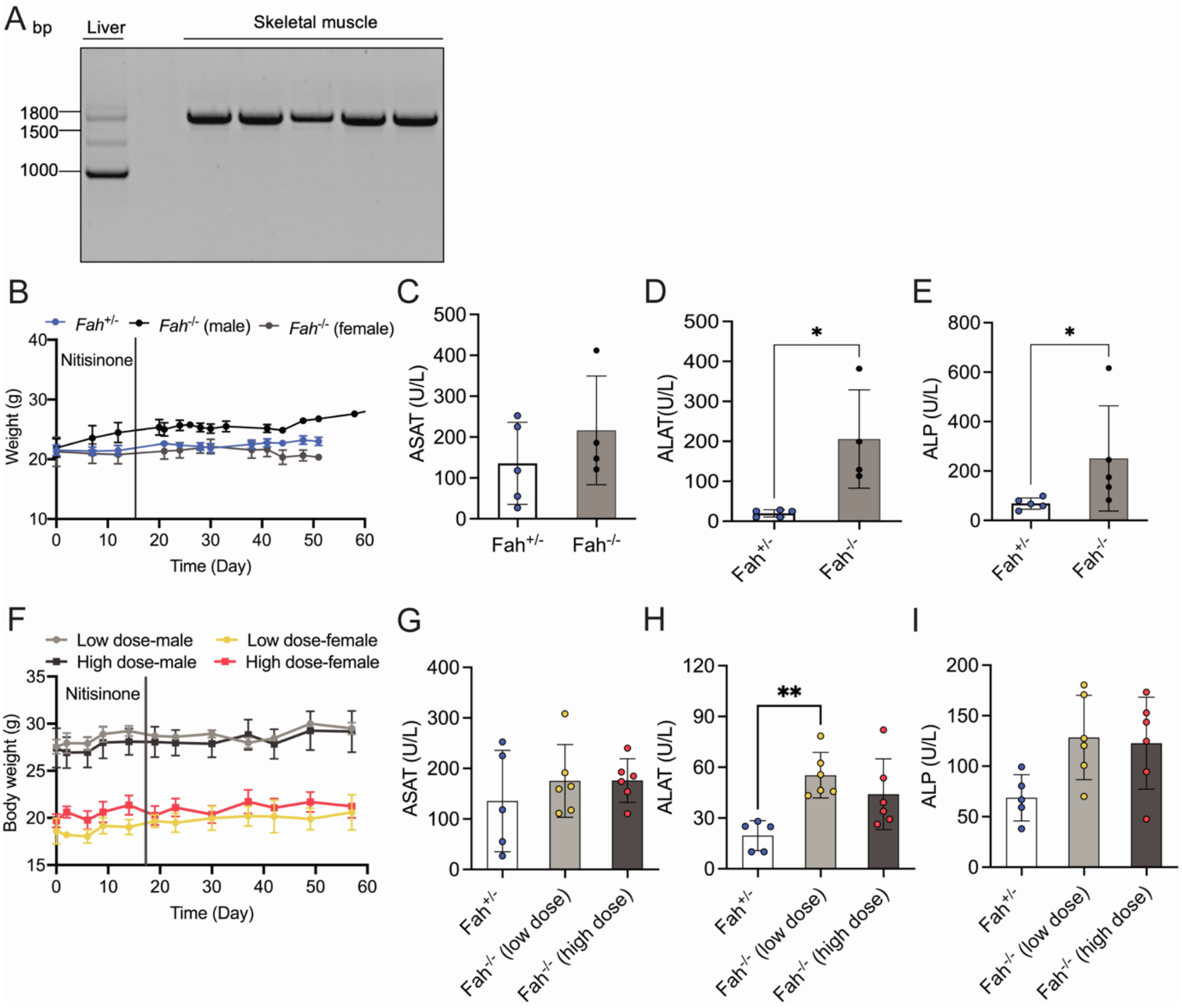

Figure S2. Safety assessment of AAV8- and LNP-mediated HPD inactivation. (A–E) AAV8-mediated *Hpd* inactivation: (A) Genomic PCR of *Hpd* in skeletal muscle (gastrocnemius muscle, n = 5). (B) Body weight changes post-injection. The left of the vertical line indicates the period during which mice had access to nitisinone drinking water, followed by hepatocyte selection. (C–E) Serum liver enzymes including ASAT, ALAT, and ALP, in units/liter (U/L) (*Fah*$^{+/-}$ controls, n=5; *Fah*$^{-/-}$ mice at 2 months after AAV injection, n=4). (F–I) LNP-mediated *Hpd* inactivation. (F) Body weight changes post-injection. (G–I) Serum ASAT, ALAT, and ALP levels ((*Fah*$^{+/-}$ controls, n=5; *Fah*$^{-/-}$ mice with low dose LNP (0.05mg/kg), n=6; *Fah*$^{-/-}$ mice with high dose LNP (0.5mg/kg), n=6). Bars represent means ± SEM. *p<0.05, **p<0.01, according to unpaired student's *t*-test or one-way ANOVA. AAV8, adeno-associated virus 8; ALAT, alanine aminotransferase; ASAT, aspartate transaminase; ALP, alkaline phosphatase; FAH, fumarylacetoacetate hydrolase; HPD, 4-hydroxyphenylpyruvate dioxygenase; LNP, lipid nanoparticle; SLiK, somatic liver knockout.

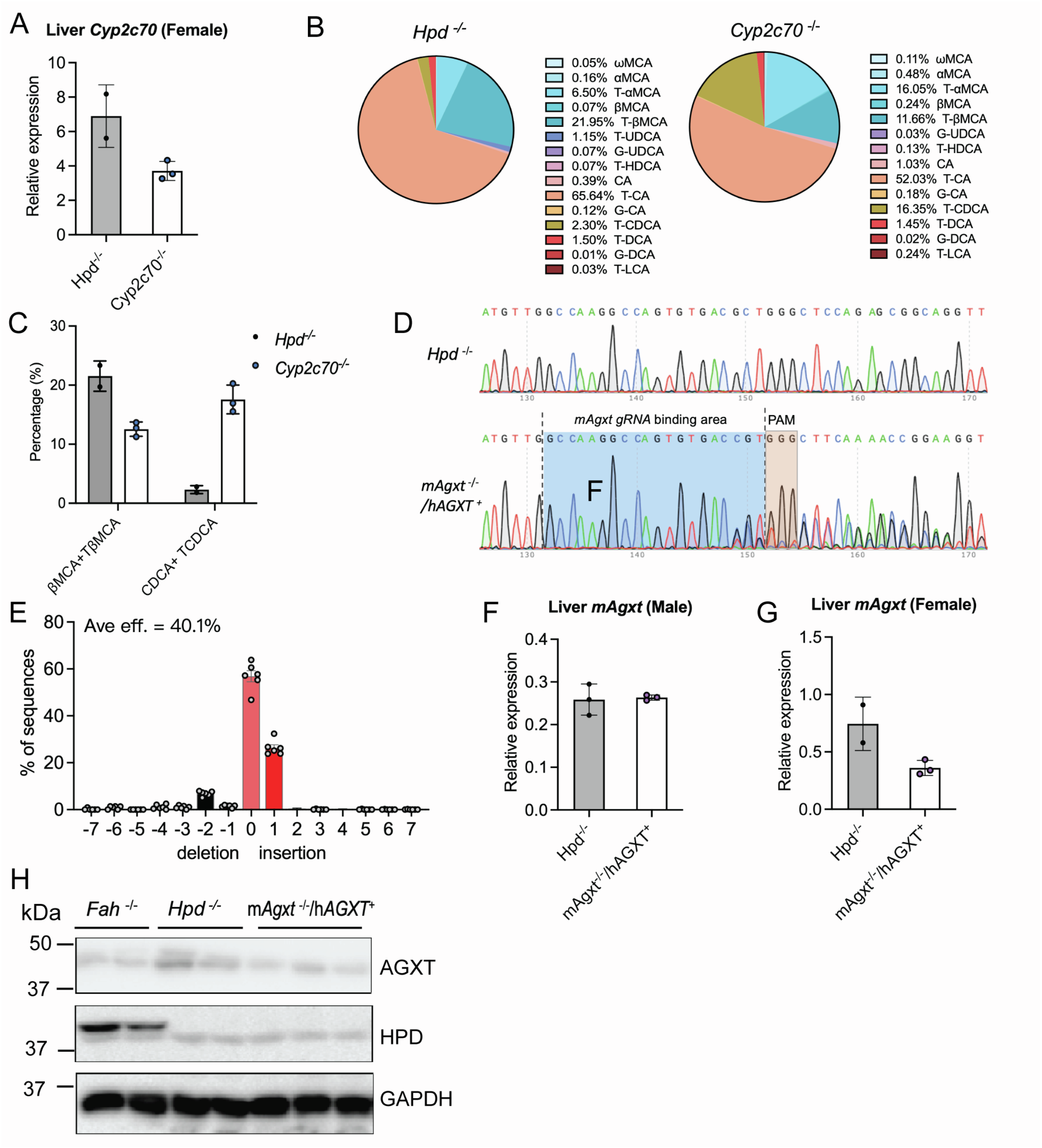

Figure S3. AAV8-SLiK mediated multiplex gene-editing. (A–C) Female mice were injected with AAV8 to knockout *Cyp2c70* (*Cyp2c70*$^{-/-}$, n=3) versus control (*Hpd*$^{-/-}$, n=2). (A) Relative mRNA expression of *Cyp2c70* in liver tissue. (B-C) Bile salt composition and quantification in bile of female mice (samples were collected 3 months post-AAV8 injection). (D-H) Mice were injected with AAV8 to knockout *Agxt* and overexpress human *AGXT*. (D) Sanger sequencing of liver genomic DNA spanning m*Agxt* gRNA target area (*mAgxt*$^{-/-}$/*hAGXT*$^{+}$, n=6) or control (*Hpd*$^{-/-}$, n=5). (E) TIDE analysis of Sanger sequencing data. (F-G) Relative mRNA expression of murine *Agxt* in liver of (F) males (*Hpd*$^{-/-}$, n=3; *mAgxt*$^{-/-}$/*hAGXT*$^{+}$, n=3) and (G) females (*Hpd*$^{-/-}$, n=2; *mAgxt*$^{-/-}$/hAGXT^{+}, n=3). (H) Western blot for HPD and AGXT in female mice (*Fah*$^{-/-}$ without AAV8 injection, n=2; *Hpd*$^{-/-}$ females, n=2; *mAgxt*$^{-/-}$ /hAGXT^{+} females, n=3). Bars represent means ± SD. Statistical significance was determined using unpaired student's *t*-test. AAV8, adeno-associated virus 8; AGXT, alanine-glyoxylate aminotransferase;

CYP2C70, cytochrome P450, family 2, subfamily c, polypeptide 70; FAH, fumarylacetoacetate hydrolase; GAPDH, glyceraldehyde 3-phosphate dehydrogenase; HPD, 4-hydroxyphenylpyruvate dioxygenase; SLiK, somatic liver knockout.

Supplemental references

1. Kunne, C, Acco, A, Hohenester, S, Duijst, S, de Waart, DR, Zamanbin, A, and Oude Elferink, RP (2013). Defective bile salt biosynthesis and hydroxylation in mice with reduced cytochrome P450 activity. *Hepatology* **57**: 1509-1517.
